# Sub- and Supercritical Extraction of Slovenian Hops (*Humulus lupulus* L.) Aurora Variety Using Different Solvents

**DOI:** 10.3390/plants10061137

**Published:** 2021-06-03

**Authors:** Katja Bizaj, Mojca Škerget, Iztok Jože Košir, Željko Knez

**Affiliations:** 1 Hmezad exim d.d., Vrečerjeva ulica 14, SI-3310 Žalec, Slovenia; k.bizaj@gmail.com; 2 Laboratory for Separation Processes and Product Design, Faculty of Chemistry and Chemical Engineering, University of Maribor, Smetanova 17, SI-2000 Maribor, Slovenia; mojca.skerget@um.si; 3 Slovenian Institute of Hop Research and Brewing, Cesta Žalskega Tabora 2, SI-3310 Žalec, Slovenia; iztok.kosir@ihps.si; 4 Department of Chemistry, Faculty of Medicine, University of Maribor, Taborska 8, SI-2000 Maribor, Slovenia

**Keywords:** hop extract, carbon dioxide, propane, sulfur hexafluoride, dimethyl ether, diffusion coefficient, bitter acids

## Abstract

This work investigates the efficiency of supercritical fluid extraction of hops with a variety of solvents including carbon dioxide (CO_2_), propane, sulfur hexafluoride (SF_6_), and dimethyl ether (DME) at various densities (low-density and high-density). Operating parameters were 50 bar, 100 bar and 150 bar and 20 °C, 40 °C, 60 °C and 80 °C for all solvents, respectively. The influence of process parameters on the total yield of extraction and content of bitter acids in the extracts has been investigated. The mathematical model based on Fick’s second law well described the experimental extraction results. Furthermore, HPLC analysis has been used to determine α- and β-acids in extracts. The yield of bitter compounds in hop extracts was largely influenced by the type of solvent, the temperature and pressure applied during extraction. The results show that CO_2_ and propane were roughly equivalent to DME in solvating power, while SF_6_ was a poor solvent at the same conditions. The highest yield as well as the highest concentration of bitter acids in extracts were obtained by using DME, where the optimal operating conditions were 40 °C and 100 bar for the extraction of α-acids (max. concentration 9.6%), 60 °C and 50 bar for the extraction of β-acids (4.5%) and 60 °C and 150 bar for the maximum extraction yield (25.6%).

## 1. Introduction

Hop (*Humulus lupulus* L.) is a dioecious, perennial climbing plant well-known for a long time due to its healing properties, bacteriostatic action and preservative qualities. It belongs to the Cannabinacea family and it is cultivated in temperate zones such as North America, West Asia and Europe [1,2]. The plant grows in excess of 6–7 m per season, producing a large amount of biomass [3]. The leaf and the steam material of the plant present approximately 75% of biomass produced by hop [4]. The harvested portion of the plant so called female inflorescence or hop cones are the most commonly used for various purposes in beverages and herbal medicine. In the cones of the female hop plant (termed lupulin glands) a range of specific bioactive secondary metabolites, including prenylated flavonoids (xanthohumol and desmethylxanthohumol), bitter acids, phenolic compounds and essential oils are found [5]. Substances found in hop plants are commonly called resins.

For centuries hops have been famously known as a key ingredient of beer, but its medicinal properties have been studied over the last decades, when scientists all over the world started exploring the effects of healing plants. Several in vitro and in vivo studies have shown that hop flowers act as antifungal agents [6] and are used to relieve the symptoms of insomnia and stress [7]. Certain hop compounds also have the potential of becoming novel anticancer agents. The essential oil derived from hops is a mixture of more than 1000 compounds, including alcohols, terpenes, organic acids and phenolics [8,9]. Polyphenols have been studied as natural additives with antimicrobial and antioxidant properties.

Presently, 90% of global hop production is used by the brewing industry as a stabilising agent and to add bitterness and aroma to beer [10]. In particular, the hop’s resins and essential oils are responsible for its characteristic aroma [11,12]. However, whilst brewing, it is crucial to prevent oxidation, once α-acids oxidize, they can no longer be isomerized into iso-α-acids leading to an unpleasant aroma and decreased bittering potential. One solution is to use hop extracts, which have many advantages. Extracts are more homogeneous than hop powder or hop pellets, have improved hop utilization, better bitterness control, improved stability on storage and reduced transport and storage costs [13].

Bitter acids are usually classified as α-acids and β-acids. α-acids are humulone (35–70% of total α-acids), cohumulone (20–65% of total α-acids) and adhumulone (10–15% of total α-acids). The substances known as β-acids include lupulone (30–55% of total β-acids), colupulone and adlupulone [14,15,16]. Both the α- and β-acids are very prone to oxidation and chemical deterioration. To prevent this, proper storage of hops is critical, requiring an oxygen barrier and refrigeration between 0 °C and 5 °C [17].

Levels of α- and β-acids in hop products depend on the hop variety, climate, soil, environmental factors at harvest and cultivation conditions [18,19,20]. In commercial use, the hop products are divided in seven groups: hop powder/pellets, enriched hop powder/pellets, speciality hop powder/pellets (made by mixing hop powder/pellets with other substances intended to improve stability), hop extract, isomerized hop products and hop oil [21]. Several scientific papers deal with the sub- and supercritical extraction of hop, using various gases as extracting solvents. The first example dates back to the late 1970s describing the use of CO_2_ in liquid or supercritical form. With respect to the choice of appropriate solvent and extraction procedure, several modifications have been reported. Unconventional solvents, such as noble gases and their mixtures are opening new perspectives, but latter requires separation processes for removing potentially harmful solvent from the final product [22]. Those solvents need to be tightly controlled with respect to their residual levels. As solvents and their residues in extract have possible harmful effects for humans, less attention has been paid to them and there is less literature data about their use for extraction of hops under sub- and supercritical conditions. However, selection of a supercritical fluid is crucial for the development of efficient supercritical fluid extraction (SFE) process.

Restrictions in the use of organic solvents, easy penetration of organic matter, and high solvating power, has meant that carbon dioxide has begun to move from some marginal applications to becoming the major solvent used for SFE processes. More than 90% of SFE studies have been performed with CO_2_ as the solvent [23]. Supercritical CO_2_ is a good solvent for the extraction of non-polar compounds such as hop soft resins, oil and aroma components, alcohols, aldehydes, olefins, paraffins, esters, amines, aromates, ketones, amides and nitrites, but its large quadrupole moment also enables it to dissolve some moderately polar compounds such as polyphenols [23,24,25]. Representatives of a large group of biologically active prenylflavonoids can also be present in the plant material. However, a cosolvent is required to extract these polar compounds.

Supercritical fluids such as Freon-22, nitrous oxide and hexane have been considered to extract polar compounds, but their applications are limited due to environmental and safety considerations. Superheated water has certain advantages such as higher extraction ability for polar compounds and products obtained in this way are solvent-free, however, it is not suitable for thermally labile compounds [26]. Several studies have also proposed compressed propane as a viable non-polar solvent and have shown good extraction yields and high antioxidant activity of extracts [27,28,29,30,31,32].

DME is known as a low-temperature and non-toxic solvent and to the best of our knowledge, there are no reports in the literature using this solvent as an extraction agent for hops. DME is partially miscible with water, which is an advantage not shared by other solvents such as propane and CO_2_. This allows processing of an aqueous feed stream and thus the need for drying of wet feed material is avoided. This represents a great advantage in hops industry where drying is costly and/or involves processing conditions that may degrade the material and alters the amount and chemical composition of the hop oils. DME is not a greenhouse gas and does not cause ozone depletion, also removing the solvent from the final product is simple and virtually complete [33].

Conversely, SF_6_ is an extremely potent and persistent greenhouse gas, it is non-flammable, and non-toxic, and considered as one of the heaviest known gases (its density is approximately five times higher than that of air). To date, only a few studies have been published that investigate its use as an extraction solvent. Phase equilibrium studies of vegetable oil–SF_6_ systems showed that as a high-pressure solvent SF_6_ shows higher solvent power for vegetable oils than CO_2_. Therefore, SF_6_ opens new perspectives as a high-pressure liquid solvent [34,35]. Depending on the extraction conditions and technologies, different compositions of acids in the final product can be obtained [36]. Aurora is an aroma variety bred from the English variety Northern Brewer and Slovenian genetic hop germ-plasm. This variety is recognised for its excellent agronomic traits in hop production and processing. Aurora is known for its pleasant hop aroma and bitterness, which offers excellent brewing value.

The aim of our study was to determine efficiencies of sub- and supercritical extraction of Aurora variety Slovenian hops using fluids of different polarity. Hence, DME was selected as the polar solvent, and CO_2_, propane and SF_6_ were used as non-polar solvents. Under identical operating conditions the solvents were in different states. CO_2_ was a liquid at experimental temperatures lower than 40 °C while at 40 °C and higher it was supercritical. Propane and DME were liquid over the entire experimental range, while SF_6_ was liquid at temperatures lower than 60 °C and supercritical at 60 °C and higher. After performing all experiments with all solvents, the difference in concentrations of α- and β-acids in obtained extracts from hop pellets was observed. The mass transport coefficients, which are crucial for the design and scale-up of the extraction process, were also determined.

Extraction experiments were performed using a semi-continuous apparatus at pressures of 50 bar, 100 bar and 150 bar, and at temperatures of 20 °C, 40 °C, 60 °C and 80 °C. The content of α- and β-acids in extracts was determined by high-performance liquid chromatography (HPLC) using a UV/VIS detector set at a wavelength of 314 nm.

## 2. Results and Discussion

### 2.1. Extraction Kinetics Study

**Figure 1:**
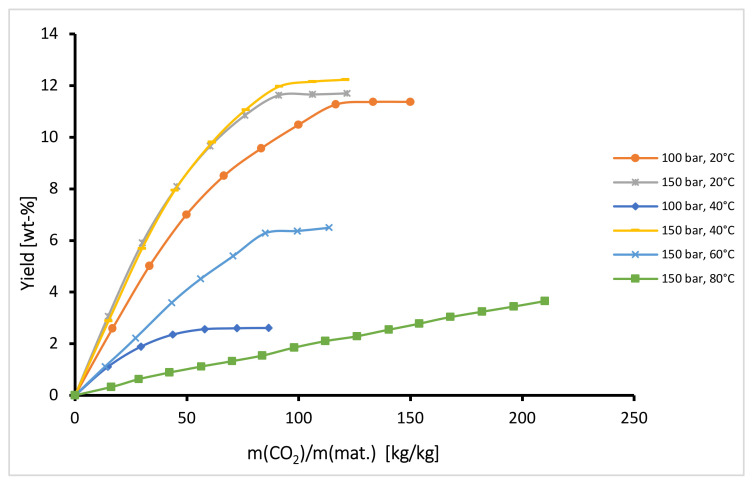
Kinetics of semi-continuous extraction of hops with dense CO_2_.

Semi-continuous operation mode was applied for extraction of oil from hops using different solvents under the same operating conditions. This technique, also termed semi-batch or semi-flow, is a process in which one phase flows continuously through a vessel containing a batch of another phase. Extensive physical contact between the solvent and the bed of hop particles was thus achieved. In the extractor, the oils in the hops were dissolved in the solvent and the target product (hop extract) was trapped in the precipitation unit. The extraction yield was calculated as the mass of the oil extracted by the mass of raw material fed into the extractor. Extraction kinetic curves for extraction of oil from hops with dense CO_2_ at different operating conditions are presented in Figure 1 (data given in Table 1 and Table 2). The obtained results can be explained by considering the density of the fluid and diffusivity of solute in the fluid. At constant temperature, the density and the solvent power of the fluid increase with increasing pressure, while the diffusivity decreases. Conversely, at constant pressure the density decreases with increasing temperature while the diffusivity increases. From Figure 1 it can be observed generally that at constant temperature the extraction yield and extraction rate increase with increasing pressure from 100 bar to 150 bar, while at constant pressure the total yield and extraction rate decrease with increasing temperature from 40 °C to 80 °C. This is in accordance with the variation of density of CO_2_ and its solvent power with temperature and pressure. This behavior is similar to that obtained by Campos et al. [37] and del Valle et al. [38] for the extraction of *Calendula officinalis* and *Humulus lupulus* L., respectively. An exception is observed at 150 bar and temperatures between 20 °C and 40 °C, where the course of both isotherms is similar in the stage of constant extraction rate while in the stage of decreasing extraction rate the yield and also extraction rate are somewhat higher at 40 °C. This is probably a consequence of higher diffusivity and higher vapor pressure of solute at higher temperature. These phenomena were also perceived by Vargas et al. [39], Zancan et al. [40] and Sovová et al. [41] for extraction of *Carqueja* essential oil, *Zingiber officinale* Roscoe and *Piper nigrum L*., respectively. The highest yield (12.2%) was obtained at 40 °C and extraction process required approximately 100 kg CO_2_/kg hops. The yields were similar to those obtained by del Valle et al. [38] for Nugget variety (13.9% at 200 bar and 50 °C) and data obtained by Langezaal et al. [42] and Kupski et al. [21] under similar conditions. At 80 °C and 150 bar, the yield was very low as a result of the weak solvent power at lower densities.

**Figure 2:**
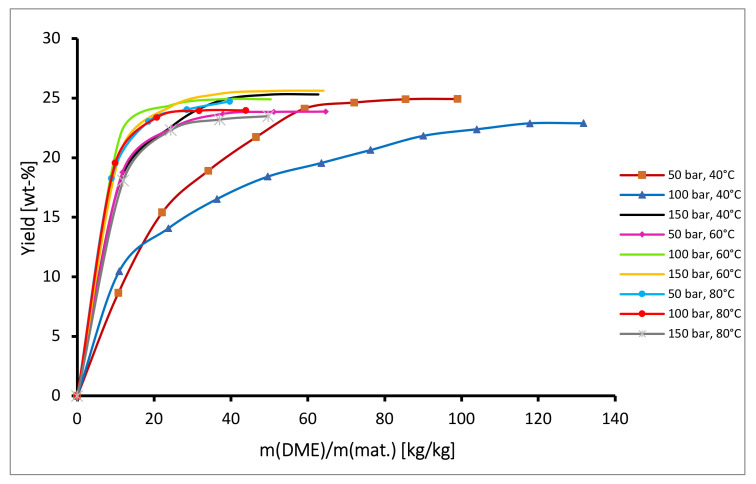
Kinetics of semicontinuous extraction of hops with DME.

In Figure 2, the kinetics of semi-continuous extraction of oil from hops with DME at different operating conditions is presented. By comparing Figure 1 and Figure 2 it can be seen, that in the case of DME the total yields were generally much higher than in the case of CO_2_ and ranged from 23 to 26%, while solvent consumption was much lower than for CO_2_, and at the maximum yield of 25.6% (150 bar and 60 °C) it was only approximately 40 kg DME/kg hops. Furthermore, the course of the isotherms at temperatures ranging from 60 °C to 80 °C and pressures from 100 bar to 150 bar is similar, indicating that at these conditions, temperature and pressure have little influence on the extraction rate and extraction yield.

**Figure 3:**
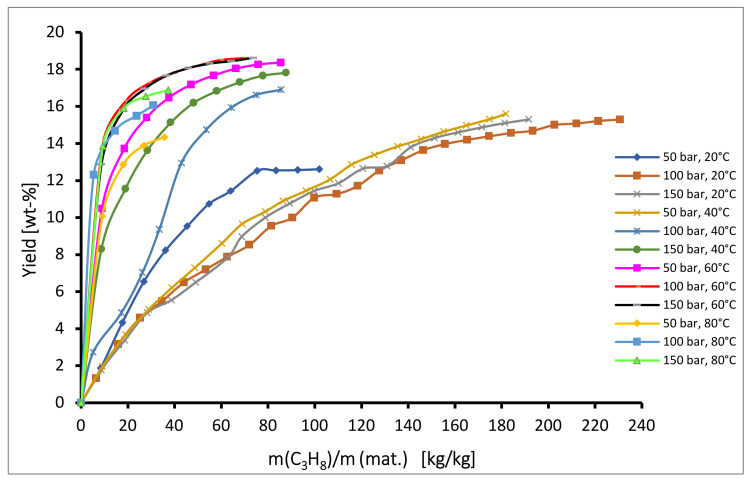
Kinetics of semi-continuous extraction of hops with propane.

When using propane as solvent, the total yields ranged between 13% to 19%, shown in the Figure 3. At 20 °C, extraction rates and extraction yields were generally low over the entire pressure range, and rates decreased with increasing pressure from 50 bar to 100 bar and stayed approximately constant with further pressure increases. At higher temperatures, the opposite trend was observed. At 40 °C the extraction rates and extraction yields increased with increasing pressure over the pressure range, while at 60 °C and 80 °C the rates and yields increased with increasing pressure from 50 bar to 100 bar and stayed approximately constant with further pressure increases. At constant pressure the extraction rates and extraction yields generally increased with increasing temperature to 60 °C and afterwards decreased with further increasing temperature to 80 °C. The max. yields at all investigated pressures were obtained at 60 °C and the values at 100 bar and 150 bar were similar and the highest (18.7%). It was noted that propane yielded higher extraction rates than carbon dioxide. These results are similar to a study by Veiga et al. [43] showing a positive effect of temperature and a negative effect of pressure on the extraction yield of Brazilian Mantiqueira hops with compressed propane (the highest yield was obtained at 60 °C and 100 bar and it was 6.0 wt-%).

**Figure 4:**
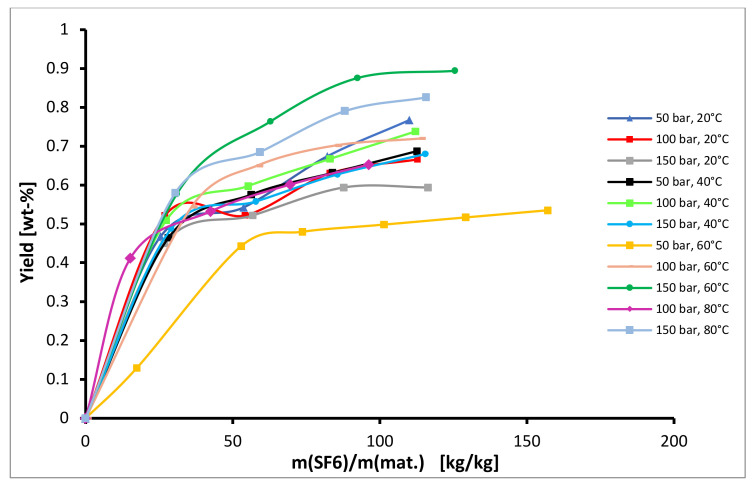
Kinetics of semi-continuous extraction of hops with SF_6_.

When extracting with SF_6_ as solvent the total yields were minimal, ranging only between 0.5% and 0.9%. The highest yield was obtained at 60 °C and pressure 150 bar, as can be seen from Figure 4. It is unclear why SF_6_ was so inefficient as a solvent, and clearly density plays a role, however further experiments may be required to understand why in this case it was worse by a factor of 10.

### 2.2. Mathematical Modelling of Kinetic Curves

Extraction kinetic curves for hop by the solvents investigated were analysed by a two-site kinetic model, that considers the presence of two parallel diffusion processes inside the solid; one faster, where the solute is transferred from the surface of the solid particle to the bulk of solvent and one slower, where the effective diffusion of solute inside the pores of solid particle is controlling the extraction rate. In between the two is a transition area where both processes can affect the extraction rate. In later stages of the extraction, only the second term on the right hand of Equation (5) remains significant, while in earlier stages of the extraction, the second exponential term is close to unity.

When modelling, the value of $m_{0}$ was set equal to the maximum extraction yield achieved by specific solvent, i.e., $m_{0\;}$ for CO_2_ was 13 mg/g; for propane 20 mg/g; for DME 26 mg/g and for SF6 1 mg/g.

The adjustable parameters obtained from best fitting of experimental curves for both extraction rate periods and deviation of the model from the data (AARD) are presented in Table 1.

For CO_2_ the experimentally obtained kinetic curves could be described by a one-extraction rate constant model, while for other solvents the experimental data were fitted by a two-rate constant model. Modelling was performed by Microsoft Excel using Solver, so the objective function (AARD) was minimized by adjusting the estimated parameters.

**Figure 5:**
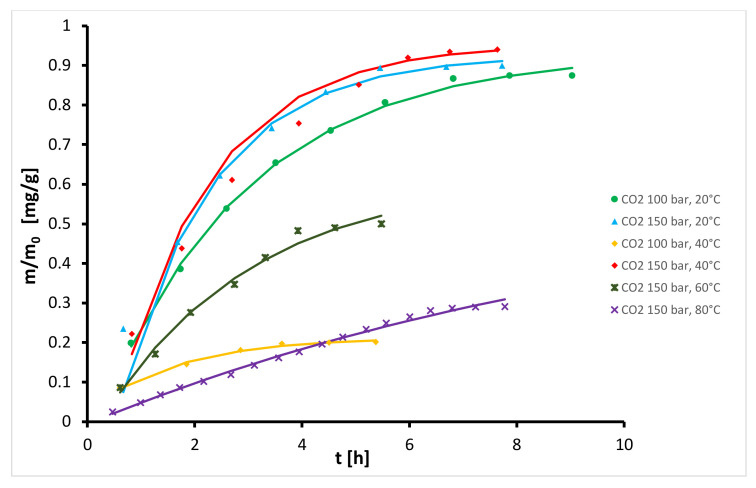
Experimental (symbols) and modelled (lines) kinetic curves for the extraction with CO_2_ as solvent.

**Figure 6:**
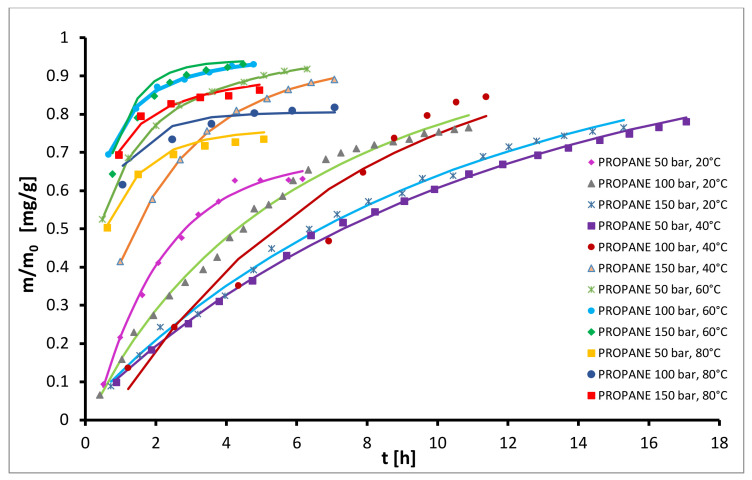
Experimental (symbols) and modelled (lines) kinetic curves for extraction with propane as solvent.

**Figure 7:**
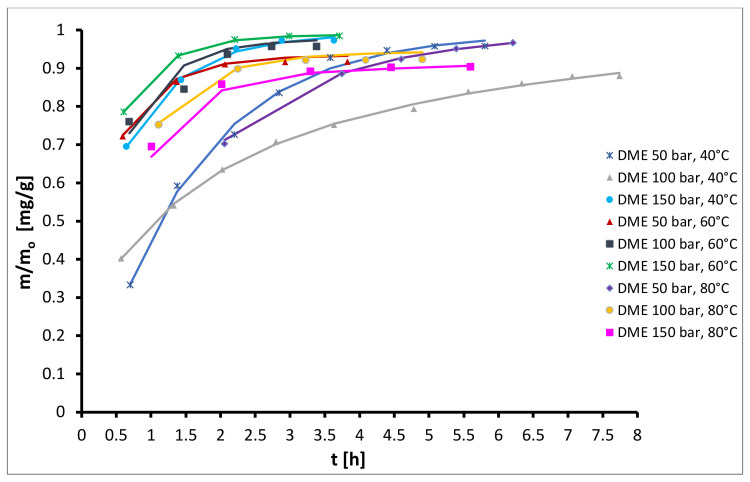
Experimental (symbols) and modelled (lines) kinetic curves for extraction with DME as solvent.

**Figure 8:**
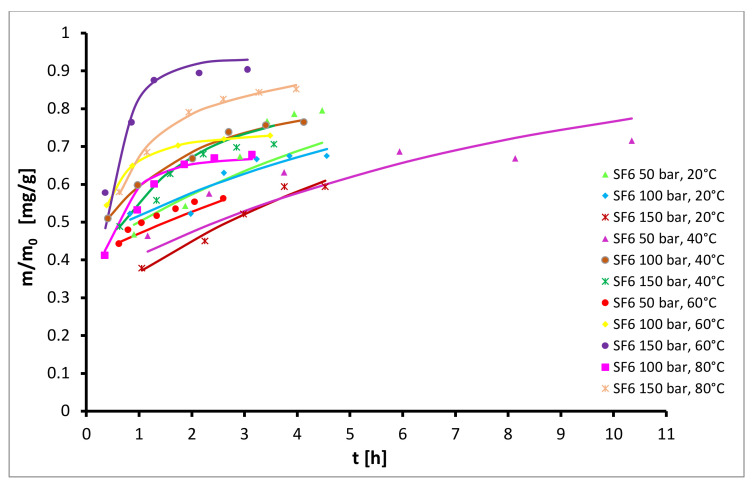
Experimental (symbols) and modelled (lines) kinetic curves for extraction with SF_6_ as solvent.

The agreement of calculated and experimental extraction curves can be observed in Figure 5 for CO_2_, in Figure 6 for propane, in Figure 7 for DME and in Figure 8 for SF_6_, respectively.

Overall, the mathematical model could fit adequately the extraction curves during all stages. AARD values, presented in Table 1, were from 0.06% to 9.10%, except for liquid propane at 100 bar and 40 °C where the AARD was 13.05%. Extraction kinetic curves were analysed for both, fast and slow extraction rate periods and diffusion coefficients for both stages *D_1_* and *D_2_* were calculated.

For CO_2_, where one extraction rate period was considered by the model, results show that the diffusion coefficient at a constant pressure of 150 bar increases with decreasing temperature from 0.039 × 10^−7^ (m^2^/s) at 80 °C to 0.248 × 10^−7^ (m^2^/s) at 20 °C. This behavior also corresponds to density variation of solvent which decreases with increasing temperature and therefore the solvent power of CO_2_ is decreased.

For extraction using propane as compressed solvent the effect of pressure is less notable than the effect of temperature. The propane is in the compressed liquid state over the entire experimental range, and the changing in density with the variation of pressure is less conspicuous. It can be noticed that extraction with propane is much faster than that with CO_2_ although both act as selective solvents for the extraction of non-polar compounds. The main reason for the temperature effect over the extraction performance is that by increasing the temperature, the density of the solvent decreases, reducing the solvation capacity. On the other hand, by increasing the pressure at a constant temperature in a subcritical state, propane diffusion into the matrix is influenced by the increase in its density and viscosity.

High extraction rate was observed at 60 °C and 100 bar and 60 °C and 150 bar where it is obvious that the process is controlled by diffusion from the particle surface. Also the fraction of solute extracted in the first period (0.651 at 100 bar and 0.640 at 150 bar) was higher than in the second period, where the diffusion of solute inside the pores of solid particles to the particle surface is controlling the extraction rate.

By using DME as extraction solvent, much higher rates of extraction and superior yields were obtained. The highest extraction rate was observed at 60 °C and 150 bar where also the highest yield was obtained. The diffusion coefficient *D_1_* at this condition was 0.736 × 10^−7^ (m^2^/s) and was higher than *D_2_* obtained for the second period which was 0.329 × 10^−8^ (m^2^/s).

From Figure 4 it can be observed the extraction with SF_6_ was not efficient and very low yields, lower than 0.9%, were obtained at all experimental conditions. The highest diffusion coefficient *D_1_* was obtained at 60 °C and 150 bar, where also the highest yield was observed.

### 2.3. HPLC Analysis of Hop Extracts

**Figure 9:**
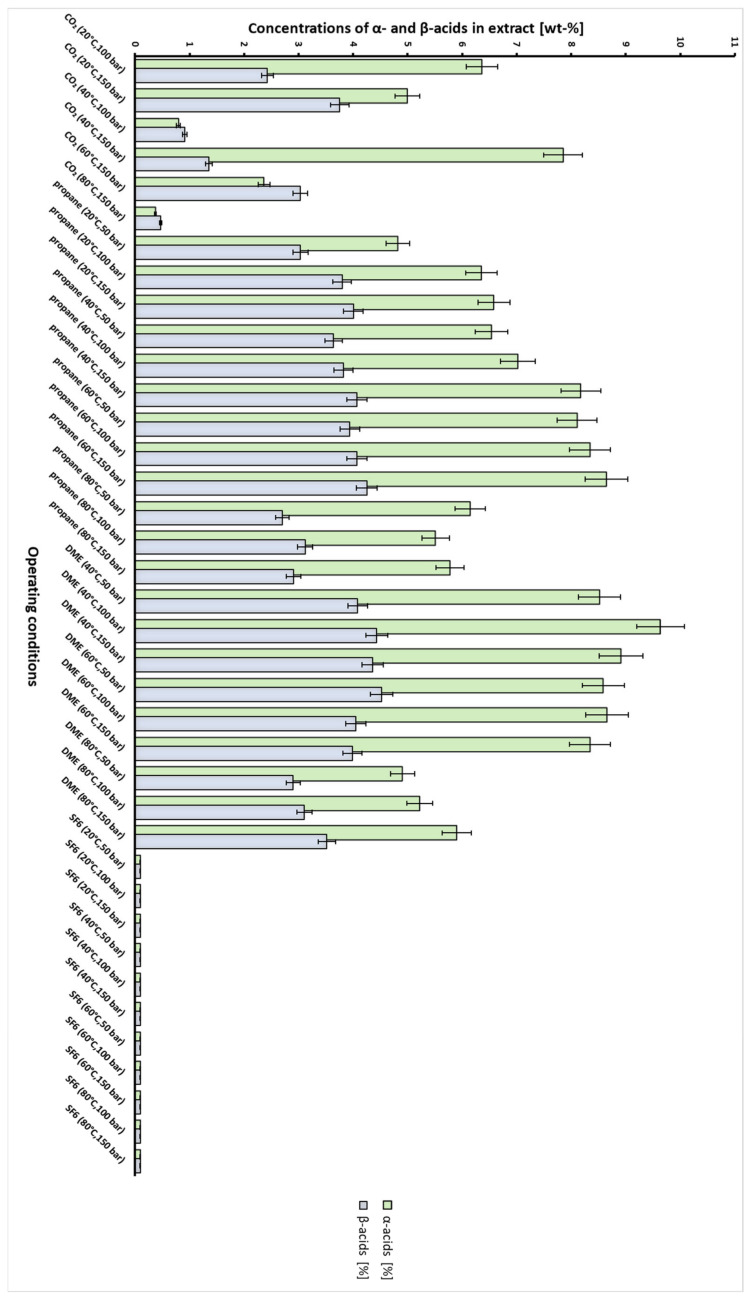
Weight fraction of α- and β-acids in extracts obtained by semicontinuous extraction of hops with dense gases at different operating conditions with standard deviation (SD) values.

The concentrations of α-acids and β-acids in hop extracts obtained with CO_2_, propane, SF_6_ and DME are presented in Table 2 and Figure 9. The yields of α- and β-acids represent the wt-% of initial acids in raw materials that were extracted by solvent and were calculated using Equation (4). The results show that the concentration of both acid groups in extract was low in the case of SF_6_. In the case of CO_2_ and propane, the optimal concentrations of α and β acids in extracts were obtained at condition, that also gave max. yield of extract. By using propane, the max. yield was higher as for CO_2_ and also the concentration of both acid groups, especially that of β acids in propane extract was higher as in CO_2_ extract (1.1 times higher for α and 3.15 times higher for β acids). In the case of DME, the highest extraction yields were obtained, however concentrations of acids in extracts were generally similar to propane extracts. When comparing the results for all solvents, the greatest concentration of α acids (9.6%) was obtained in the extract derived by DME at 40 °C and 100 bar which also presented a high yield (22.9%). The highest concentration of β acids (4.5%) was also obtained by DME, however at 60 °C and 50 bar with a yield of 23.9%. Based on these results it can be concluded that the best solvent for the isolation of α and β acids is DME, as it gives the highest extraction yields and the highest concentrations of acids in extracts.

**Figure 10:**
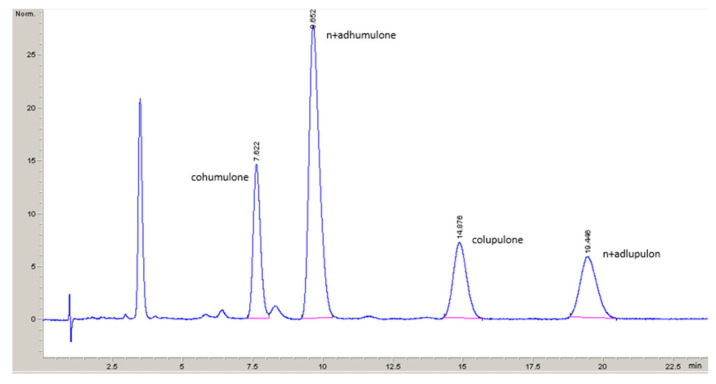
HPLC analysis chromatogram specimen of Aurora variety hop essential oils.

The extraction efficiency of α-acids and β-acids as indicated by the acid yield was the highest in the case of DME, with yields higher than 95% for both acids at specific conditions were obtained, followed by propane, where the max. yield was 87.6% for α-acids and 90.5% for β-acids, respectively. In the case of CO_2_ the yields of both acids were around 79.7% at specific conditions while for SF_6_, the yields were below 0.01%. Chromatogram specimen of Aurora variety hop essential oils derived from HPLC analysis can be seen in Figure 10.

## 3. Conclusions

This study demonstrates various non-conventional extractions of Aurora variety Slovenian hops (*Humulus lupulus* L.). The solvents were selected based on their differing polarity. CO_2_, propane and SF_6_ were used as non-polar solvents and DME as a polar solvent. The best extraction was achieved using DME at 60 °C and 150 bar, with a global yield of 25.6%. DME also showed a high variability with temperature and pressure both having a remarkable effect on the extraction yield and also on max. concentration of both acids. The highest concentration of α-acids was 9.6% (40 °C, 100 bar) and β-acids 4.5% (60 °C, 50 bar). In this study, propane was the second-best solvent regarding extraction yield (18.7% under operating conditions 60 °C and 150 bar) and the concentrations of both α- and β-acids were also the highest (8.7% of α-acids and 4.3% of β-acids). With CO_2_ the maximum yield (12.2%) was reached at 40 °C and 150 bar with a max. concentration of α-acids at 7.9%. SF_6_ proved to be a very poor solvent for extracting hop resins, with a maximum extraction yield of only 0.9% at 60 °C and 150 bar. The extraction kinetics were modelled by the two-site kinetic model and good agreement between experimental data and model predictions was observed with percent average absolute relative deviation in the range of 0.06% to 13.05%. The challenges to be faced using DME for further development of the process are around regulatory acceptance and overcoming safety issues concerning the flammability.

## 4. Materials and Methods

### 4.1. Materials

The hop pellets used in this study were supplied by Hmezad exim d.d. (Žalec, Slovenia). The cones of the hop cultivar Aurora were collected in Slovenia between the 23 and 30 August, air dried at 60 degrees and compressed into pellets. These were then purged with inert gas N_2_ and sealed into laminated polythene/metallised polyester bags. Pellets were stored out of sunlight in storage between +1 and +4 °C to prevent excessive oxidation and oxidation of hop resins and essential oils. The composition of hop resins in raw material (pellets) was: total α-acids 9.9% and total β-acids 4.7%, determined according to Analytica-EBC 7.7 method [44].

CO2 (>99.5% purity) was obtained from Messer Slovenija d.o.o. (Ruše, Slovenia). Propane (>99.5% purity) and DME (>99.5% purity) were obtained from Linde plin d.o.o. (Celje, Slovenia). SF_6_ (>99.5% purity) was purchased from Istrabenz d.d. (Koper, Slovenia). All chemicals used for HPLC analysis were purchased from Sigma Aldrich (Taufkirchen, Germany).

### 4.2. Equipment and Experimental Procedures

#### 4.2.1. Methods for Characterization of Material

The pellets were ground in a domestic grinder after being in refrigerated storage for a month. Sieve analysis was performed to separate the fractions and to obtain homogeneous particles. The median particle size of ground hop pellets, determined from particle size distribution curves (frequency and cumulative arithmetic) was 1300 µm. The moisture content of extracts and raw material was determined using standard Analytica–EBC method 7.2 (Analytica–EBC 2007) [45]. The moisture content was considered in all calculations so yields and concentrations are expressed on a dry basis.

#### 4.2.2. Sub- and Supercritical Fluid Extraction (SFE)

**Figure 11:**
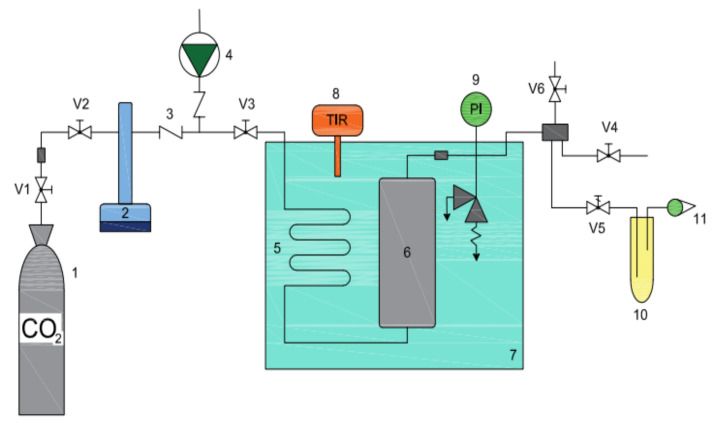
Schematic diagram of the high-pressure extraction apparatus (1—gas tank, 2—high pressure pump, 3—check valve, 4—HPLC pump, 5—gas heating tube, 6—autoclave, 7—water tank with heater, 8—temperature regulator, 9—manometer pressure gauge, 10—extract, 11—rotameter, V1–V6—valves).

The experiments using different solvents were performed by a semi-continuous flow apparatus [46] (Figure 11). The maximum operating pressure and temperature of apparatus were 200 bar and 100 °C, respectively. The extractor (V = 60 mL) was charged with approximately 45 g of ground material. The temperature of the water bath was regulated and maintained at a constant value (±0.5 °C; LAUDA DR. R. Wobster GmbH & Co. KG, Lauda Königshofen, Germany). The apparatus was purged first with nitrogen and later with the gas used for extraction. Liquefied gas was continuously pumped with a high-pressure syringe pump (Pmax = 200 bar; ISCO, Louisville, KY, USA) through the preheating coil and over the bed of sample in the extractor. The flow rate of solvent, measured at room temperature and atmospheric pressure, was approximately 0.20 kg/h, respectively. The solvent flow rate was measured with a flow meter (Elster Handel GmbH, Mainz, Germany). The product precipitated in the separator (glass tube), where the separation was performed at 1 bar and at a temperature of 20 °C. The product collected in the glass tube was weighed (±0.1 mg) and yield was calculated. The density of the gas inside the extractor (ρ) was obtained from the NIST Chemistry WebBook, for CO_2_, propane and SF_6_ [47]. The density of DME was calculated with Peng-Robinson equation of state using the Aspen Plus process simulation software.

#### 4.2.3. High-Performance Liquid Chromatography (HPLC) Analysis of Extract

According to Analytica–EBC 7.7 method [44] high performance liquid chromatography (HPLC) was employed to determine the α- and β-acids in extracts with liquid chromatograph Agilent 1200 Series (Agilent, Palo Alto, CA, USA). 0.5 g of hop extract was diluted with 50 mL methanol and 5 mL of this solution was additionally diluted in 50 mL of methanol. Extracts were filtered through disposable syringe filters, Chromafil Xtra PET-45/25 (Macherey-Nagel, Düren, Germany) and 10 µL injection loop on HPLC injector was used. The separation was achieved on Nucleodur 5-100 C18, 125 × 4 mm HPLC analysis column (Macherey-Nagel). Isocratic mobile phase constituted from distilled water, methanol (J.T. Baker, Phillipsburg, NJ, USA) and 85% aqueous solution of orthophosphoric acid (Merck, Darmstadt, Germany) in a ratio of 775/210/9 (v/v/v) was used and the detection was carried out with a diode array detector (DAD) set at 314 nm. The quantification was done by the external standard ICE4 (NATECO2, Wolnzach, Germany). All solvents were of analytical grade purity.

The content of individual fractions (α- and β-acids) in the extract was calculated by the following equation:

(1) $$c_{iv}=\frac{m_{s}c_{is}A_{iv}}{m_{v}A_{is}}$$

where $c_{iv}$ stands for the concentration of component i in sample expressed in weight %, $m_{s}$ is the weight of the calibration standard in grams, $c_{is}$ is the concentration of component i in standard, $A_{iv}$ is the peak area of component i in the sample, $m_{v}$ is weight of the sample and $A_{is}$ means surface of the peak i of calibration standard. The content of α-acids is calculated as the sum of the two fractions: cohumulone (coh), nhumulone and adhumulone together (nh + adh); and the content of β-acids as the sum of the two fractions: colupulone (col), nlupulone and adlupulone together (nl + adl):

(2) $$c_{\alpha }=c_{\left(coh\right)}+c_{\left(nh+adh\right)}$$

(3) $$c_{\beta }=c_{\left(col\right)}+c_{\left(nl+adl\right)}$$

The concentrations of α- and β-acids are expressed as the W % of acids determined in the extract and the efficiency of extraction is expressed in grams of acids per 100 g of material.

Yields of α- and β-acids which represent the wt-% of discharged acids in relation to the initial concentration of acids in raw material are calculated using Equation (4):

(4) $$Yield_{\left(\alpha ,\beta -acids\right)}=\frac{m_{\left(\alpha ,\beta -acids\right)in\;extract}}{m_{\left(\alpha ,\beta -acids\right)in\;raw\;material}}*100\%$$

#### 4.2.4. Mathematical Model

Supercritical extraction processes are based on diffusion. The solvent must diffuse into the matrix, and the extracted material diffuse out of the matrix into the solvent. The extraction kinetics were modelled by the so-called “two-site kinetic model”. The model is an extension of the “one-site kinetic model”, mostly referred as Crank’s hot ball diffusion model, which is based on Fick’s second law of diffusion [48,49]. Experimental extraction curves were analysed considering a fast and a slow extraction period relevant to two different solute fractions. One fraction of the solute is quickly released and another fraction in the matrices is slowly released. This two-stages diffusion mechanism can be written for the ratio of mass of analyte removed after time t to the initial mass, $m_{0}$, consisting of two first-order expressions, as follows:

(5) $$\frac{m_{t}}{m_{0}}=1-k_{1}\exp\left(-k_{2}t\right)-k_{3}\exp\left(-k_{4}t\right)$$

in which $m_{t}$ is the mass of the analyte removed by the extraction fluid after time t (mg/g), $m_{0}$ the total initial mass of analyte in the matrix (mg/g), $m_{t}/m_{0}$ the fraction of the solute extracted after time t, a certain fraction ($k_{1})$ desorbs at a fast rate determined by $k_{2}$ and the other fraction ($k_{3})$ defined by $k_{4}$ which desorbs at a slower rate [50,51,52]. It is important to know that this model relies solely on time and it does not include any factor describing extraction flow-rate [53].

The Microsoft Excel Solver regression routine was used to fit the extraction data to Equation (5). The fit parameters were $k_{1}$, $k_{2}$, $k_{3}$ and $k_{4}$, as previously described.

The model presented by Equation (5) is practically the same as diffusion model for solid-liquid extraction of spherical particles proposed by Crank [54] (Equation (6)):

(6) $$\frac{m_{0}-m}{m_{0}}=\frac{6}{\pi^{2}}\left[f_{1}exp\left\{-\frac{\pi^{2}D_{1}}{R^{2}}t\right\}+f_{2}exp\left\{-\frac{\pi^{2}D_{2}}{R^{2}}t\right\}\right]$$

where R is the sphere particles’ radius and $f_{1}$ and $f_{2}$ are the fractions of the solute, which are extracted with diffusion coefficients $D_{1}$ and $D_{2}$, respectively [48]. Therefore, from results obtained by Equation (5) for constants $k_{2}$ and $k_{4}$ diffusion coefficients $D_{1}$ and $D_{2}$ were calculated.

The average absolute relative deviation, for all experimental data, was calculated using the following expression:

(7) $$AARD\;\left(\%\right)=\frac{100}{N}\sum_{i=1}^{N}\frac{\left|yield_{calc}-\left.\;yield_{exp}\right|\right.}{yield_{exp}}$$

## Figures and Tables

**Table 1:** Model adjustable parameters for extraction of hops with respect to experimental conditions.

Solvent	T [°C]	*p* [bar]	ρ * $\left[\mathrm{kg}/m^{3}\right]$	$k_{1}$ ** [/]	$k_{3}$ ** [/]	$D_{1}\times 10^{7}$ *** $\left[m^{2}/s\right]$	$D_{2}\times 10^{8}$ *** $\left[m^{2}/s\right]$	AARD **** [%]
CO_2_	20	100	856.3	0.533	0.075	0.155	/	1.66
	20	150	904.0	1.255	0.074	0.248	/	0.88
	40	100	628.6	0.184	0.786	0.252	/	1.99
	40	150	780.2	1.254	0.046	0.243	/	7.77
	60	150	604.1	0.673	0.385	0.153	/	5.19
	80	150	427.2	0.619	0.387	0.039	/	4.09
Propane	20	50	510.8	0.767	0.318	0.216	0.006	2.51
	20	100	521.3	0.161	0.852	0.220	0.566	3.38
	20	150	530.2	0.012	0.957	0.299	0.417	3.48
	40	50	481.2	0.007	0.963	0.379	0.384	1.81
	40	100	495.5	0.007	1.095	0.413	0.630	13.1
	40	150	506.9	0.739	0.134	0.202	0.032	0.46
	60	50	446.4	0.391	0.260	0.449	0.817	0.33
	60	100	467.2	0.651	0.241	0.537	0.681	0.35
	60	150	482.1	0.640	0.063	0.547	0.059	3.56
	80	50	400.9	0.414	0.262	0.405	0.063	1.81
	80	100	435.2	0.637	0.326	0.422	0.011	2.96
	80	150	455.6	0.606	0.327	0.516	0.422	1.38
DME	40	50	630.3	1.059	0.008	0.291	0.116	1.62
	40	100	635.3	0.332	0.428	0.344	0.745	0.70
	40	150	639.8	0.627	0.037	0.545	1.270	0.53
	60	50	592.7	0.894	0.129	0.726	0.196	0.81
	60	100	599.7	1.259	0.057	0.729	0.445	3.00
	60	150	606.5	0.927	0.027	0.736	0.329	0.11
	80	50	549.1	1.379	1.090	0.624	0.205	0.38
	80	100	560.1	0.885	0.073	0.628	0.209	1.13
	80	150	571.2	0.987	0.126	0.657	0.234	1.35
SF_6_	20	50	1469.7	0.936	0.024	0.067	0.586	8.90
	20	100	1545.8	0.657	0.251	0.073	0.200	4.14
	20	150	1598.0	0.810	0.394	0.078	0.286	2.65
	40	50	1273.4	0.935	0.148	0.056	0.063	9.10
	40	100	1419.9	0.501	0.438	0.294	0.239	1.20
	40	150	1495.4	0.695	0.416	0.312	0.205	3.60
	60	50	753.3	0.935	0.042	0.051	0.043	3.62
	60	100	1269.2	0.577	0.508	0.936	0.162	0.06
	60	150	1383.9	0.996	0.078	0.966	0.162	4.90
	80	100	1085.7	0.740	0.571	0.813	0.050	3.30
	80	150	1263.1	0.510	0.297	0.789	0.831	1.14

* Density of solvent obtained from NIST Chemistry WebBook^47^ at constant temperature (*T*) and pressure (*P*). Density for DME was obtained from Aspen Plus using Peng-Robinson EOS. ** Fractions desorbed at a fast rate (*k*_1_) and desorbed at a slower rate (*k*_3_). *** Diffusion coefficients $D_{1}$ and $D_{2}$ ($m^{2}$/s). **** Average absolute relative deviation (%).

**Table 2:** Content of α and β-acids in extract expressed in weight % (mean ± SD) and the yield of acids. (Limit of quantification LoQ = 0.1%).

Solvent	T [°C]	P [bar]	Yield * [%]	$Yield{\;}_{\left(\alpha -acids\right)}^{**}$ [%]	$Yield{\;}_{\left(\beta -acids\right)}^{**}$ [%]	$W{\;}_{\left(\alpha -acids\right)}^{***}$ [%]	$W{\;}_{\left(\beta -acids\right)}^{***}$ [%]
CO_2_	20	100	11.4	64.5	48.3	6.4 ± 0.3	2.4 ± 0.1
	20	150	11.7	50.7	79.8	5.0 ± 0.2	3.8 ± 0.2
	40	100	2.6	8.1	19.5	0.8 ± <0.1	0.9 ± <0.1
	40	150	12.2 ± 0.2 a	79.7	29.6	7.9 ± 0.4	1.4 ± < 0.1
	60	150	6.5	24.3	64.4	2.4 ± 0.1	3.0 ± 0.1
	80	150	3.7	3.8	10.1	0.4 ± <0.1	0.5 ± < 0.1
Propane	20	50	12.6	48.8	64.7	4.8 ± 0.2	3.0 ± 0.1
	20	100	15.3	64.5	80.7	6.4 ± 0.3	3.8 ± 0.2
	20	150	15.3	66.7	85.2	6.6 ± 0.3	4.0 ± 0.2
	40	50	15.6	66.2	77.4	6.5 ± 0.3	3.6 ± 0.2
	40	100	16.9	71.2	81.2	7.0 ± 0.3	3.8 ± 0.2
	40	150	17.8	82.9	86.7	8.2 ± 0.4	4.1 ± 0.2
	60	50	18.4	82.2	83.6	8.1 ± 0.4	3.9 ± 0.2
	60	100	18.6	84.6	86.7	8.4 ± 0.4	4.1 ± 0.2
	60	150	18.7 ± 0.1 b	87.6	90.5	8.7 ± 0.4	4.3 ± 0.2
	80	50	14.3	62.3	57.4	6.1 ± 0.3	2.7 ± 0.1
	80	100	16.1	55.8	66.4	5.5 ± 0.2	3.1 ± 0.1
	80	150	16.9	58.5	61.9	5.8 ± 0.3	2.9 ± 0.1
DME	40	50	24.9	86.4	86.9	8.5 ± 0.4	4.1 ± 0.2
	40	100	22.9	97.6	94.2	9.6 ± 0.4	4.4 ± 0.2
	40	150	25.3	90.3	92.8	8.9 ± 0.4	4.4 ± 0.2
	60	50	23.9	87.0	96.2	8.6 ± 0.4	4.5 ± 0.2
	60	100	24.9	87.7	86.3	8.7 ± 0.4	4.1 ± 0.2
	60	150	25.6 ± 0.5 c	84.6	84.9	8.3 ± 0.4	4.0 ± 0.2
	80	50	24.7	49.7	61.7	4.9 ± 0.2	2.9 ± 0.1
	80	100	24.0	52.9	66.0	5.2 ± 0.2	3.1 ± 0.1
	80	150	23.5	59.9	74.8	5.9 ± 0.3	3.5 ± 0.2
SF_6_	20	50	0.8	0.01	<0.01	0.1 ± <0.01	<0.1 ± <0.01
	20	100	0.7	<0.01	<0.01	<0.1 ± <0.01	<0.1 ± <0.01
	20	150	0.6	0.01	<0.01	0.1 ± <0.01	<0.1 ± <0.01
	40	50	0.7	0.01	<0.01	0.1 ± <0.01	<0.1 ± <0.01
	40	100	0.7	0.01	<0.01	0.1 ± <0.01	<0.1 ± <0.01
	40	150	0.7	<0.01	<0.01	<0.1 ± <0.01	<0.1 ± <0.01
	60	50	0.5	0.01	<0.01	0.1 ± <0.01	<0.1 ± <0.01
	60	100	0.7	<0.01	<0.01	<0.1 ± <0.01	<0.1 ± <0.01
	60	150	0.9 ± 0.01 d	<0.01	<0.01	<0.1 ± <0.01	<0.1 ± <0.01
	80	100	0.7	<0.01	0.01	<0.1 ± <0.01	<0.1 ± <0.01
	80	150	0.8	0.01	0.01	0.1 ± <0.01	0.1 ± <0.01

* Extraction yield expressed in wt%, a, b, c, d—based on triplicate experiments (mean ± standard deviation). ** Yield of α- and β-acids isolated regarding initial concentration of acids in raw material expressed in wt%. *** Concentration of α- and β-acids in extract expressed in wt% (mean ± standard deviation).
